# Hemophagocytic lymphohistiocytosis complicating invasive pneumococcal disease: a pediatric case report

**DOI:** 10.1186/s12887-020-1915-7

**Published:** 2020-01-13

**Authors:** Mitsuru Tsuge, Machiko Miyamoto, Reiji Miyawaki, Yoichi Kondo, Hirokazu Tsukahara

**Affiliations:** 1 Department of Pediatrics, Okayama University Graduate School of Medicine, Dentistry, and Pharmaceutical Sciences, 2-5-1 Shikata-cho, Kita-ku, Okayama, 700-8558 Japan; 2 Department of Pediatrics, Matsuyama Red Cross Hospital, 1 Bunkyo-cho, Matsuyama, Ehime 790-8524 Japan; 3 Department of Pediatrics, Ehime University Graduate School of Medicine, Shitsukawa, Toon, Ehime 791-0295 Japan

**Keywords:** Child, Hemophagocytic lymphohistiocytosis, Invasive pneumococcal disease, Pneumococcal conjugate vaccine, Serotype replacement

## Abstract

**Background:**

Hemophagocytic lymphohistiocytosis (HLH) is an infrequent but life-threatening disease due to excessive immune activation. Secondary HLH can be triggered by infections, autoimmune diseases, and malignant diseases. *Streptococcus pneumoniae* is a pathogenic bacterium responsible for invasive pneumococcal disease (IPD) such as meningitis and bacteremia. Although the pneumococcal conjugate vaccine (PCV) has led to reductions in IPD incidence, cases of IPD caused by serotypes not included in PCV are increasing. There are few reports of secondary HLH caused by IPD in previously healthy children. We herein report a rare case of a previously healthy boy with secondary HLH complicating IPD of serotype 23A, which is not included in the pneumococcal 13-valent conjugate vaccine (PCV-13).

**Case presentation:**

An 11-month-old boy who had received three doses of PCV-13 was hospitalized with prolonged fever, bilateral otitis media, neutropenia and elevated C-reactive protein (CRP) levels. Blood culture on admission revealed *S. pneumoniae*, leading to a diagnosis of IPD. HLH was diagnosed based on a prolonged fever, neutropenia, anemia, hepatosplenomegaly, hemophagocytosis in the bone marrow, and elevated serum levels of triglycerides, ferritin, and soluble interleukin-2 receptor. He received broad-spectrum antibiotics and intravenous immunoglobulins for IPD and high-dose steroid pulse therapy and cyclosporine A for HLH; thereafter, his fever resolved, and laboratory findings improved. The serotype of the isolated *S. pneumoniae* was 23A, which is not included in PCV-13.

**Conclusions:**

It is important to consider secondary HLH as a complication of IPD cases with febrile cytopenia or hepatosplenomegaly, and appropriate treatment for HLH should be started without delay.

## Background

Hemophagocytic lymphohistiocytosis (HLH) is an infrequent but potentially life-threatening hematological disorder associated with an excessive systemic inflammatory response [1, 2]. It is caused by uncontrolled activation and proliferation of macrophages, lymphocytes, and dendritic cells, resulting in hemophagocytosis and cytokine storm. HLH can occur as a primary disorder, caused by a genetic mutation, or as secondary sporadic cases triggered by infection, autoimmune diseases, immunodeficiency, or malignant diseases. Viruses are the most common cause of infection-associated HLH, with Epstein Barr virus (EBV) being the most common trigger. Particularly in children, virus-associated HLH accounts for 94% of infection-associated HLH, and approximately half of the virus-associated HLH cases are caused by EBV [3]. Bacterial, fungal, parasitic, and tropical infections can also trigger HLH; however, there are very few reports of bacterial-associated HLH in children.

*Streptococcus pneumonia* is an important pathogen causing invasive infections such as pneumonia, meningitis, and bacteremia, leading to high morbidity and mortality. The pneumococcal conjugate vaccine (PCV) has led to dramatic reductions in cases of invasive pneumococcal disease (IPD) worldwide, including Japan; however, IPD cases caused by serotypes not included in the 13-valent pneumococcal conjugate vaccine (PCV-13) are becoming increasingly frequent due to serotype replacement [4, 5].

Secondary HLH caused by *S. pneumoniae* has been previously described as a complication of IPD in immunodeficient children; however, there are few reports in previously healthy children. We describe a rare case of a previously healthy boy with secondary HLH caused by IPD due to *S. pneumoniae* 23A, which is not included in the PCV-13.

## Case presentation

An 11-month-old, previously healthy boy with a 1-day history of fever was presented to his family physician. Blood tests revealed leukocytosis (10,000 /μL) and an elevated C-reactive protein (CRP) level (5.0 mg/dL). His family physician suspected that he was suffering from mild pneumonia, and he was administered a single intravenous dose of ceftriaxone sodium and discharged on daily suppository antibiotics (ceftizoxime sodium) and then sent home. However, his fever persisted, so he was taken to consult the family physician every day. His family physician advised that he continue using antibiotics; however, progressive neutropenia and an increased CRP level were shown. He was therefore referred to our hospital.

His parents were not consanguineous, and there were no cases of immunodeficiency or HLH in the patient’s family. He had no remarkable medical history and his physical growth and development had been normal. He had been fully immunized with three doses of PCV-13. A physical examination on admission revealed that his temperature was 41.1 °C, blood pressure was 88/58 mmHg, heart rate was 202 beats/min, respiratory rate was 60/min, and SpO_2_ was 100%. He showed a normal growth and development. He had conjunctival pallor, dried lips, reddened pharyngeal mucosa, bilateral inflamed eardrums, hepatosplenomegaly, and petechiae on his extremities. The blood tests revealed leukopenia (1570 /μL), neutropenia (63 /μL), increased CRP (37.8 mg/dL), increased procalcitonin (PCT) (97 mg/dL), increased aspartate aminotransferase (AST) (287 IU/L), increased lactate dehydrogenase (LDH) (3474 IU/L), increased fibrin/fibrinogen degradation products (FDP) (1257 μg/mL), hyperferritinemia (26,500 ng/mL), hypertriglyceridemia (389 mg/dL), and increased soluble IL-2 receptor (sIL-2R) (4400 U/mL) **(**Table 1**)**. A blood test on the second day of admission showed anemia (7.8 g/dL). Serum electrolytes, blood urea nitrogen, blood sugar, and serum creatinine levels were within normal range. The results of the urinalysis was normal. Leukocytosis was absent in the cerebrospinal fluid (CSF), and the concentrations of protein and glucose in the CSF were within the reference range (31 mg/dL and 53 mg/dL, respectively). There was no evidence EBV on serology or DNA in whole blood. The immunoglobulin M titers for cytomegalovirus, human herpesvirus-6, and measles were also negative. The results of immunochromatographic antigen tests for influenza virus in nasal discharge, adenovirus in throat swab, rotavirus in stool, human metapneumovirus in nasal discharge, and group A Streptococcus in throat swab were negative. The β-D-glucan level was also negative.

**Table 1:** Serial changes in inflammatory parameters during invasive pneumococcal disease with hemophagocytic lymphohistiocytosis

Days of Hospitalization (reference range)	WBC (/μl) (3500–9000)	NEC (/μl) (1240–4610)	Hgb (g/dl) (13.6–18.3)	PLT (×10^4^/μl) (14.0–37.9)	CRP (mg/dl) (0–0.5)	PCT (ng/ml) (< 0.5)	AST (IU/l) (10–40)	LDH (IU/l) (120–245)	FDP (μg/ml) (< 5)	Ferritin (pg/ml) (38–150)	Urinary β2MG (ng/ml) (0–289)	sIL-2R (U/ml) (145–519)	IL-6 (pg/ml) (0–149)	IL-10 (pg/ml) (< 8)	IL-18 (pg/ml) (< 100)	IFN-γ (pg/ml) (< 2)	sCD163 (pg/ml) (< 100)
-4	10,000	4330	11.1	20.1	5.0	ND	ND	ND	ND	ND	ND	ND	ND	ND	ND	ND	ND
−3	8900	3222	10.7	22.9	7.1	ND	ND	ND	ND	ND	ND	ND	ND	ND	ND	ND	ND
−2	5900	1080	11.1	20.1	7.9	ND	ND	ND	ND	ND	ND	ND	ND	ND	ND	ND	ND
−1	4200	0	9.5	13.4	6.5	ND	ND	ND	ND	ND	ND	ND	ND	ND	ND	ND	ND
0	1570	63	9.1	17.5	37.8	97	287	3474	1257	26,500	59,247	4400	5366	82	646	34.5	618
2	700	7	7.8	13.8	34.3	77	187	2537	114	16,950	59,488	6946	399	109	1928	12.3	531
4	5690	2902	11.6	20.3	17.5	13	89	979	17	4170	193,960	3601	103	17	838	10	460
7	36,130	20,594	9.1	18.4	3.3	1.2	39	617	14	385	295	1623	11	5.9	457	7.1	253
10	23,330	13,298	9.5	48.0	1.4	0.8	52	494	6.1	213	245	994	7.7	4.2	316	7.1	189
14	13,240	3972	8.3	61.0	0.2	0.7	30	312	2.8	107	122	372	6.8	3.8	206	7.1	115
17	8300	1909	7.9	54.7	0.1	0.8	27	247	2.3	62	95	349	4.0	2.8	87	7.1	62

*WBC* White blood cell count, *NEC* Neutrophil count, *Hgb* Hemoglobin, *PLT* Platelet count, *CRP* C-reactive protein, *PCT* Procalcitonin, *AST* Aspartate aminotransferase, *LDH* Lactate dehydrogenase, *FDP* Fibrin/fibrinogen degradation products, *β2MG* Beta-2 microglobulin, *sIL-2R* Soluble interleukin-2 receptor, *IL* Interleukin, *ND* No data

Computed tomography revealed hepatosplenomegaly without pleural effusion, ascites, or abscess. Cerebral magnetic resonance imaging revealed fluid retention in the bilateral mastoid processes. Echocardiography revealed no pericardial effusion, valve vegetations, or coronary artery dilation. To differentiate other causes of neutropenia and anemia, bone marrow puncture was performed, revealing that the number of nucleated cells was decreased (2.0 × 10^4^/μL), and the ratio of myeloid to erythroid precursor cells was increased to 9.29. Phagocytosis of erythroblasts by macrophages was observed. After submitting blood cultures, meropenem hydrate was started on admission. We diagnosed him with severe IPD with a rapid progressive deterioration in his general condition and a severe systemic inflammatory response; therefore, intravenous immunoglobulin was also administered as an add-on antimicrobial therapy. In addition, he was diagnosed with HLH based on prolonged fever; neutropenia; anemia; hepatosplenomegaly; hemophagocytosis in the bone marrow; and elevated serum levels of triglycerides, ferritin, and sIL-2R.

On the second day of hospitalization, *S. pneumoniae* was isolated from blood cultures drawn on admission. Cultures from urine and CSF samples were negative. We explained to the patient’s parents that the prompt treatment for HLH with immunomodulatory therapy using steroid and immunosuppressants was needed, and that these treatments might have an adverse effect on the severity of the IPD; thereafter, we obtained a consent for treatment from the patient’s parents. On the same day, high-dose methylprednisolone (30 mg/kg/day, 3 days) was started. Prednisolone (1.5 mg/kg/day) and cyclosporin A (6 mg/kg/day) were started after the methylprednisolone pulse; thereafter, white blood cell count, neutrophil count, and platelet counts gradually increased while the levels of AST, LDH, FDP, ferritin, and CRP returned to their normal ranges. The minimum inhibitory concentration (MIC) of penicillin G for the detected *S. pneumoniae* was 0.5 μg/mL; therefore, it was identified as penicillin-intermediate *S. pneumoniae* (PISP). On the fourth day of hospitalization, his fever resolved. He was then switched to cefotaxime sodium (150 mg/kg/day) as de-escalation, although the MIC of cefotaxime was not relatively good (0.5 μg/mL). Blood culture tests on the 4th, 8th, and 11th days of hospitalization were negative. Cefotaxime sodium was discontinued on the 14th day of hospitalization, whereas prednisolone and cyclosporin A were discontinued on the 55th day of treatment. He was discharged without any complications on the 22nd day of hospitalization. There was no recurrence of HLH and no developmental delay up to when he was 3 years old.

The pneumococcal capsular serotype of *S. pneumoniae* isolated from the patient’s blood was identified as 23A, which was not included in PCV-13. Considering the possibility of primary immunodeficiency, immunological function tests for the number and function of T cells, B cells, serum complement titers, serum immunoglobulin titers, granulocyte phagocytic function, and natural killer cell activity were performed and found to be within the normal range (19.4% assay at effector-to-target ratio of 20:1; standard value 14.1–48.7%). We did not test the immunoglobulin G (IgG) titer for *S. pneumoniae* in this patient; however, the IgG titer for pertussis was positive, confirming the reaction of the vaccine that he had previously received. In addition to inflammatory parameters such as CRP, PCT, ferritin, and urinary β2-microglobulin (β2-MG), we measured serum cytokines such as sIL-2R, interleukin (IL)-2, IL-4, IL-6, IL-10, IL-18, interferon-γ (IFN-γ), tumor necrosis factor-α (TNF-α), and soluble CD163 (sCD163) serially using the enzyme-linked immunosorbent assay**.** The levels of CRP, PCT, ferritin, sIL-2R, IL-6, IL-10, IL-18, IFN-γ, and sCD163 in the serum were highly peaked at admission and then gradually decreased. However, the levels of urinary β2-MG remained elevated on 3rd day of hospitalization even after high-dose steroid pulse therapy and then decreased rapidly after the induction of cyclosporin A on the 5th day of hospitalization. The levels of IL-2, IL-4, and TNF-α were below the detection limit.

## Discussion and conclusions

The incidence of pediatric HLH due to bacterial infections in Japan was reported to be about 6% of the infection-associated HLH cases [6]. Several studies have reported bacterial infection-associated HLH [7], including *Mycoplasma pneumoniae* [8, 9], *Listeria monocytogenes* [10], Leptospira [11], Brucella [12], group B streptococcus [13], and *Coxiella burnetii* [14]. Secondary HLH caused by *S. pneumococcus* has been previously described in adults after splenectomy or with primary immunodeficiency [15, 16]. However, there are very few cases of secondary HLH complicated with IPD in previously healthy children.

In this case, *S. pneumoniae* was identified in two sets of blood cultures; therefore, we considered that IPD could have triggered HLH. Considering that our patient also had bilateral otitis media, we hypothesized that it was the source of the blood infection by *S. pneumoniae*. Unfortunately, tympanostomy and culture were not performed. The Clinical and Laboratory Standards Institute criteria for MICs of *S. pneumoniae* in bacteremia were used to classify isolates as penicillin-susceptible (≤ 0.06 μg/ml), penicillin-intermediate (0.12–1 μg/ml), or penicillin-resistant (≥ 2 μg/ml). Based on these criteria, the *S. pneumoniae* detected in this case was identified as PISP. Although the MIC of cefotaxime was not very good (0.5 μg/ml), we were able to perform de-escalation successfully using cefotaxime sodium at high-dose level.

We also considered the possibilities of other viral or bacterial infections. Blood tests and immunochromatographic antigen tests revealed that the possibility of being infected with EBV, cytomegalovirus, human herpesvirus-6, measles, influenza virus, adenovirus, rotavirus, human metapneumovirus, and group A streptococcus was less likely; however, we did not test an exhaustive list of all the possible pathogens which can cause secondary HLH. There was also the possibility of primary HLH in this case; one limitation of this case report is that we did not perform perforin staining, a granule release assay, or a genetic analysis to determine the causes of primary HLH. We are planning these further analyses for the differentiation of familial hemophagocytic lymphohistiocytosis.

The initial first-line therapy for HLH involves treating the triggering cause as this can be effective enough to withdraw the immune activation and control the inflammatory cytokine storm in most patients. However, in acutely ill patients or those whose condition is deteriorating, immunomodulatory therapy such as a combination of high-dose steroid, cyclosporine A, and etoposide-based chemotherapy may be needed, according to the Histiocyte Society’s diagnostic and therapeutic guidelines for HLH (HLH-2004) [17]. Our patient also appeared unwell with a severe systemic inflammatory response; thus, we considered immunomodulatory therapy to be vital as an early intervention in the course of the disease.

Viruses are the most common cause of infection-associated HLH, and the mechanisms by which viruses contribute to HLH pathogenesis remain to be clarified. However, it is thought that viruses can increase the host’s susceptibility to HLH by modulating the host immune system via recognition by the innate or adaptive immune system, interfering with cytokine balances, and inhibiting apoptosis [18]. Serum inflammatory cytokines are known to be increased in the serum of viral HLH cases, particularly, IFN-γ, TNF-α, IL-6, IL-10, and IL-18 [19]. No report has described the changes in serum cytokine levels in cases of bacteria-associated HLH; therefore, we measured the serial changes in serum cytokines that have been reported previously in cases of virus-associated HLH. In the present case as well, the levels of IL-6, IL-10, IL-18, and IFN-γ in the serum were elevated in the acute phase, similar to the cytokine pattern reported in virus-associated HLH [20–22]. The kinetics of the cytokines in our patient were consistent with those in previous studies that analyzed patients with pediatric HLH, suggesting that the cytokine pattern in serum of HLH cases might be similar even though the susceptibility to HLH varies depending on the pathogen.

In our case, he suffered from IPD even though he had already received three doses of PCV-13; the detected *S. pneumoniae* serotype was 23A, which is not included in PCV-13. *S. pneumoniae* is a causative agent in IPD such as meningitis and bacteremia, and the incidence rate of IPD caused by the serotypes included in PCV-13 decreased after the introduction of PCV worldwide. In the United States, IPD was reduced by 75% in 2000 when PCV-7 (including serotypes 4, 6B, 9 V, 14, 18C, 19P, and 23F) was introduced, and it decreased by 90% after the introduction of PCV-13 (including the new serotypes 3, 5, 6A, 7F, and 19A) in 2010 [23, 24]. In Japan, PCV-7 was introduced in 2010, and PCV-7 was switched to PCV-13 as a routine immunization program in 2013. As a result, the incidence rate of IPD has markedly reduced [25]. However, serotype replacement has been observed several years after the introduction of PCV in Japan as well as worldwide [26, 27]. A recent study reported that IPD cases caused by serotype 23A account for approximately 7% of IPD cases in Japan [28].

In conclusion, we described a previously healthy boy with secondary HLH caused by IPD with a serotype that is not included in PCV-13. This case suggests that it is important to consider the complication of secondary HLH when children with IPD present with unusual clinical findings, such as a prolonged fever, cytopenia, and hepatosplenomegaly, even if they already received the PCV in the past or have no past medical history. Prompt diagnosis and appropriate treatment for HLH should be provided without delay.

## Data Availability

The data used in this report are available from the corresponding author on reasonable request.

## Abbreviations

AST: Aspartate aminotransferase
CRP: C-reactive protein
CSF: Cerebrospinal fluid
DNA: Deoxyribonucleic acid
EBV: Epstein Barr virus
FDP: Fibrin/fibrinogen degradation products
HLH: Hemophagocytic lymphohistiocytosis
IFN: Interferon
IgG: Immunoglobulin G
IL: Interleukin
IPD: Invasive pneumococcal disease
LDH: Lactate dehydrogenase
MIC: Minimum inhibitory concentration
PCT: Procalcitonin
PCV: Pneumococcal conjugate vaccine
PCV-13: 13-valent pneumococcal conjugate vaccine
PCV-7: 7-valent pneumococcal conjugate vaccine
PISP: Penicillin-intermediate *Streptococcus pneumoniae*
sCD163: Soluble CD163
sIL-2R: Soluble interleukin-2 receptor
SpO_2_: Saturation of percutaneous oxygen
TNF-α: Tumor necrosis factor -α
β2-MG: β2 –microglobulin
